# The role of B2M in cancer immunotherapy resistance: function, resistance mechanism, and reversal strategies

**DOI:** 10.3389/fimmu.2025.1512509

**Published:** 2025-03-21

**Authors:** Xiaowen Han, Jiayi Zhang, Weidong Li, Xiaodong Huang, Xueyan Wang, Bofang Wang, Lei Gao, Hao Chen

**Affiliations:** ^1^ Lanzhou University Second Hospital, Lanzhou, China; ^2^ Department of Surgical Oncology, Lanzhou University Second Hospital, Lanzhou, China; ^3^ Key Laboratory of Environmental Oncology of Gansu Province, Lanzhou, China

**Keywords:** B2M, cancer immunotherapy, immune resistance mechanism, expression regulation, reverse resistance strategy

## Abstract

Immunotherapy has emerged as a preeminent force in the domain of cancer therapeutics and achieved remarkable breakthroughs. Nevertheless, the high resistance has become the most substantial impediment restricting its clinical efficacy. Beta-2 microglobulin (B2M), the light chain of major histocompatibility complex (MHC) class I, plays an indispensable part by presenting tumor antigens to cytotoxic T lymphocytes (CTLs) for exerting anti-tumor effects. Accumulating evidence indicates that B2M mutation/defect is one of the key mechanisms underlying tumor immunotherapy resistance. Therefore, elucidating the role played by B2M and devising effective strategies to battle against resistance are pressing issues. This review will systematically expound upon them, aiming to provide insight into the potential of B2M as a promising target in anticancer immune response.

## 1 Introduction

Immunotherapy is a promising therapeutic modality for cancer following surgery, chemotherapy, radiotherapy, and targeted therapy. Distinct from conventional therapies, it exerts anti-tumor efficacy by activating or reconstituting immune defense system, thereby further prolonging the survival of cancer patients and enhancing their quality of life (1, 2). At present, prominent tumor immunotherapies incorporate immune checkpoint inhibitors (ICIs) (3, 4), cancer vaccines (5–7), and chimeric antigen receptor T cells (CAR-T) (8–10), which have revolutionized tumor treatment and represent a significant milestone in the field. Despite the fact that unprecedented durable response has been observed in clinical practice, the majority of patients do not respond to the treatment (primary resistance), and some responders relapse after subsequent treatment (secondary resistance) (11–13). Thus, augmenting the therapeutic potency of immunity and transcending immune resistance constitute the cardinal challenges within the contemporary sphere of tumor therapy. Against this backdrop, in-depth exploration of biomarkers related to the mechanism of tumor immunotherapy resistance is of tremendous significance. Notably, programmed cell death ligand 1 (PD-L1), cytotoxic T lymphocyte-associated protein 4 (CTLA-4), Janus kinase 1/2 (JAK1/2), signal transducer and activator of transcription 3 (STAT3), and phosphatase and tensin homolog (PTEN) have been pinpointed as key biomarkers of immune therapy resistance (14–17). Nevertheless, additional biomarkers are required to provide a scientific basis and directional guidance for enhancing the efficacy of tumor immunotherapy, reversing immune resistance, and resulting in significant amelioration in survival among oncological patients.

B2M, originally discovered as a low molecular protein in the serum of patients with renal tubular lesions (18), is predominantly synthesized by platelets, lymphocytes, and polymorphonuclear leukocytes. It is ubiquitously present in blood, urine, cerebrospinal fluid, saliva, and colostrum, albeit in trace amounts. Notably, its levels are not influenced by gender, age, or the amount of muscle tissue (19). Under physiological conditions, B2M levels in serum remain relatively stable due to constant production and efficient renal clearance. Specifically, B2M is filtered through the glomerulus and nearly completely reabsorbed by the proximal tubules (20). Therefore, abnormal elevation of B2M in serum and urine serve as sensitive indicators of proximal tubule function and glomerular filtration efficiency, making it a valuable surrogate biomarker for renal impairment (21). Moreover, serum B2M has been recognized as a biomarker for a variety of diseases, such as lymphoma, coronary artery, inflammatory, central nervous system and autoimmune diseases (20, 22, 23). Structurally, B2M is a non-glycosylated protein consisting of 119 amino acids with a molecular weight of approximately 12 kDa, encoded on chromosome 15 (15q21.1). It consists of seven antiparallel β-chains that form two β-sheets connected by a single disulfide bond, presenting in a characteristic immunoglobulin (Ig)-like β-sandwich structure (24). As an indispensable subunit (a crucial component of the light chain) of MHC-I molecule, it is entrusted with presenting endogenous tumor antigens to CTLs to elicit immune killing effects. However, emerging evidence suggests that B2M expression is frequently compromised in numerous cancers due to deficiency, mutation and epigenetic suppression (25–27), and B2M alterations are associated with low response rates. B2M mutation/defect can significantly diminish the recognition of cancer cells by lymphocytes, thereby inducing tumor immune evasion and resistance to immunotherapy. A melanoma patient with B2M defeat developed resistance subsequent to receiving PD-1 inhibitor (28), whereas a high level of B2M mRNA was linked to enhanced response to PD-1-based immunotherapy (29). These findings underscore the critical role of B2M in immunotherapy resistance and suggest its potential as a target for overcoming resistance. To date, only one review has delved into the role of B2M in cancer immunotherapy, highlighting that B2M alterations are prevalent across various cancers and are linked to tumor immune escape and immunotherapy resistance, and may serve as a potential biomarker for ICIs treatment (27). Interestingly, this review not only elucidates the biological functions of B2M, its expression regulation, mechanisms B2M-related immunotherapy resistance, and the association between B2M and immunotherapy resistance along with the reversal strategies, but also explores the correlation using emerging technologies such as single-cell RNA sequencing (scRNA-seq), imaging mass cytometry (IMC) and CRISPR/Cas9. Additionally, it was observed that in the context of B2M defect, antigen recognition by CTLs is impaired, while other immune cells (such as CD4^+^ T lymphocytes, NK cells, and γδ T cells) may still retain their ability to kill tumor, further indicating that the activation of these cells can overcome immunotherapy resistance caused by B2M deficiency.

## 2 Biological function of B2M

B2M is located on chromosome 15 and encompasses 4 exons. The sequence of B2M gene exhibits certain homology with immunoglobulin (Ig) constant region and MHC class I molecule α3 domain (30). Structurally, B2M consists of two disulfide-linked β-sheets and can form amyloid fibers under specific pathological circumstances (31). B2M is prevalently expressed in considerable nucleated cells, encompassing immune cells and tumor cells. Serum B2M is regarded as a biomarker for the severity of infections, amyloidosis, renal injury, lymphoproliferative diseases, and aging-related disorders (23, 32), while the B2M on the cell membrane surface non-covalently binds to the heavy chain of MHC-I molecule to execute diverse immune functions. The most typical one is to present tumor antigens by participating in the formation of the antigen peptide-MHC class I complex (pMHC-I) to activate CTLs and exerted effects by reinvigorating our immune system to battle against cancer.

**Figure 1:**
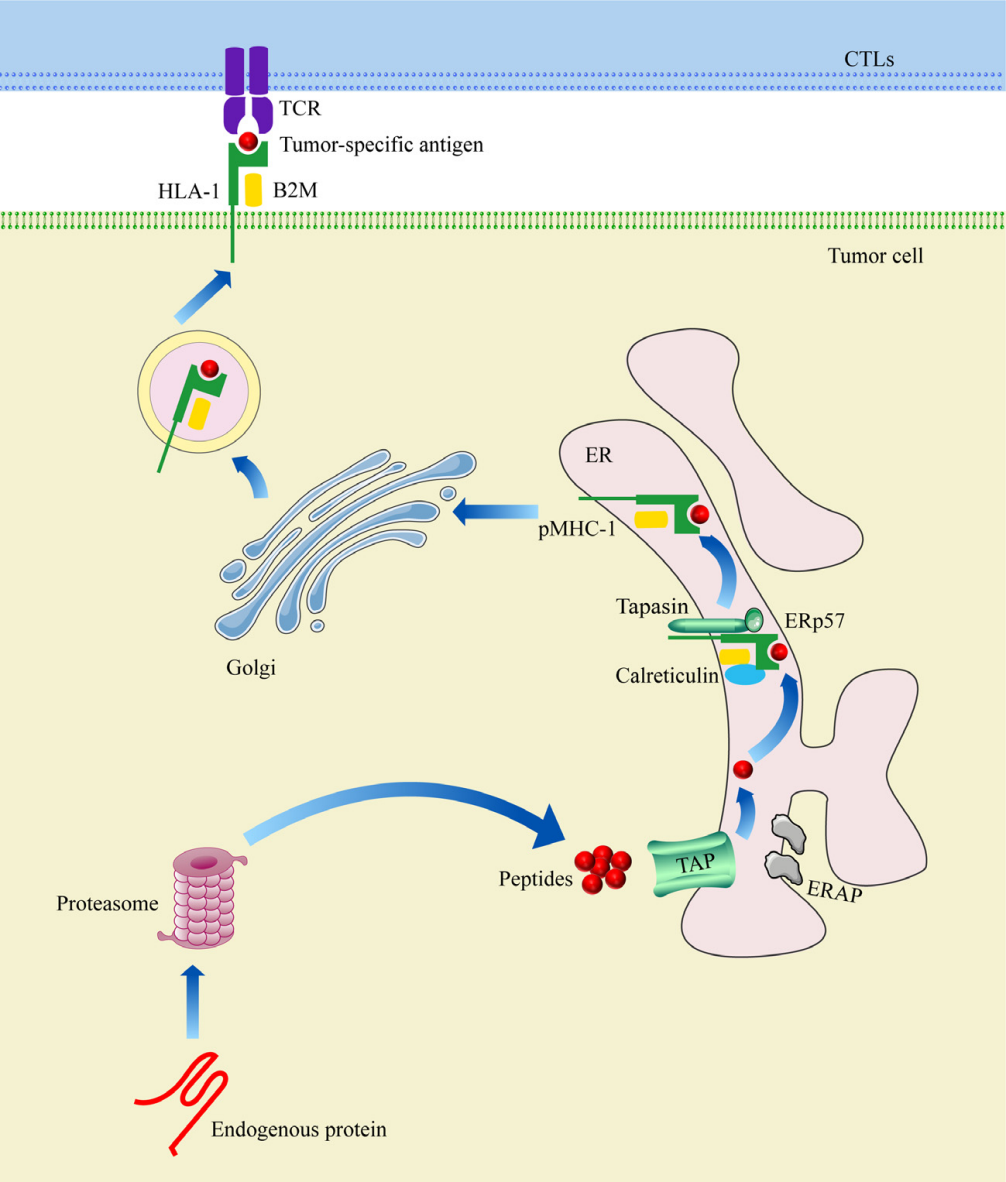
Schematic Illustration of B2M participating in MHC-I restricted tumor antigen presentation.

B2M, the light chain (β chain) of the MHC-I complex, serves as one of the most salient functions in participating in MHC-I-restricted tumor antigen presentation, including four principal steps (**Figure 1**): (1) Proteasome-mediated degradation of endogenous protein for the acquisition of antigenic peptides; (2) The conveyance of antigenic peptides to the endoplasmic reticulum (ER) by the transporter associated with antigen processing (TAP); (3) The loading of antigenic peptides into the peptide-binding groove of the MHC-I complex to constitute a stable pMHC-I complex and its translocation to the surface of tumor cells via the Golgi; (4) The recognition of the pMHC-I complex by CTLs to exert immune killing effects. Endogenous protein antigen (tumor antigen) is degraded by proteasome in the cytoplasm for the attainment of antigenic peptides. Next, antigenic peptides are transported to the endoplasmic reticulum by TAP and are pruned by endoplasmic reticulum aminopeptidase 1 (ERAP1) and endoplasmic reticulum aminopeptidase 2 (ERAP2) to acquire mature antigenic peptides consisting of 8-9 amino acids (30). In the ER, human leukocyte antigen-I (HLA-I), B2M, endoplasmic reticulum protein 57 (ERp57), Tapasin, and calreticulin collectively form a peptide-loading complex (PLC), facilitating the folding of the MHC-1α chain and obtaining mature MHC-I complex (33). Subsequently, antigenic peptides are loaded into the peptide-binding groove to form a stable pMHC-I complex, which disengages from the PLC and is transported to the surface of tumor cells through Golgi for recognition by the T cell receptor (TCR) on CTLs to eradicate tumor cells.

## 3 Regulation of B2M expression

The expression of B2M is modulated by multiple mechanisms. At the genetic level, B2M mutation facilitates tumor immune evasion through hampering antigen presentation. Currently, B2M mutation comprises point mutation, frameshift mutation, and loss of heterozygosity (LOH), which have been documented in malignant tumors like gastric cancer, colorectal cancer, renal cancer, and melanoma. In advanced melanoma, it was determined that 29.4% of patients exhibited B2M gene mutation, deletion, or LOH (34); similarly, HLA LOH was detected in 40% of non-small cell lung cancer (NSCLC) patients (35). Nucleotide-binding oligomerization domain-Like Receptor family Caspase recruitment domain containing 5(NLRC5), also known as the Class I transactivator (CITA), constitutes the CITA enhanceosome complex through binding to RFX5 and RFXAP and serves as a transcriptional activator to expedite the expression of MHC class I molecule. Yoshihama et al. (36) divulged that the methylation of the NLRC5 promoter was inversely associated with B2M expression in melanoma and engendered a diminution in MHC -I molecule expression and the advent of tumor immune escape. On the contrary, the Kobayashi team utilized a gene-specific system based on CRISPR/Cas9 technology, designated as TRED-I (Targeted reactivation and demethylation for MHC-I), to accomplish targeted demethylation of the NLRC5 promoter, induce an augmentation in NLRC5 expression, thereby upregulating MHC-I, B2M, TAP1 and genes encoding immune proteasome components (LMP2/PSMB9/β1i, LMP7/PSMB8/β5i), ultimately culminating in enhanced tumor immunogenicity (37). Consequently, NLRC5 contributes to reconstituting the expression and antigen presentation of B2M/MHC-I on tumor cells to potentiate CD8^+^ T cell dominated anticancer immunity.

In addition, the expression of B2M is also subjected to epigenetic mechanisms, encompassing DNA methylation, histone methylation, and histone deacetylation. In 2019, the research team led by Mark A. Dawson at the Peter MacCallum Cancer Centre in Australia employed whole-genome CRISPR/Cas9 screening to demonstrate that polycomb repressive complex 2 (PRC2) can silence key genes involved in the MHC-I antigen processing pathway, thereby facilitating T cell-mediated immune evasion. Furthermore, they ascertained that histone modifications like H3K4me3 and H3K27me3 enriched in the promoter region of the B2M gene inhibited the expression of MHC class I molecule (38). Grounded on the aforementioned findings, a study (39) revealed that after treating Renca cells with alpha ketoglutarate (the specific substrate of histone demethylase), the abundance of H3K4me1 and the expression level of B2M protein significantly elevated, while the abundance of H3K4me3 decreased, indicating that histone methylation might orchestrate the expression of the B2M gene. Methylation of the B2M gene promoter was observed to interrelate with transcriptional inactivation and down-regulation of B2M expression in colorectal cancer featuring microsatellite instability (MSI) (40), which was up-regulated using the DNA methyltransferase inhibitor, DNMTi. Previous studies have demonstrated that histone deacetylase inhibitor (HDACi) can reverse immune resistance by upregulating the expression of genes pertinent to antigen processing and presentation pathways (including B2M) in melanoma cell lines (41). Analogously, in clinical practice, HDACi can also enhance B2M/MHC-1 expression to address resistance to ICIs therapy (42–44).

Empirical studies have unequivocally demonstrated that the interferon-γ (IFNγ) molecule potentiates the transcriptional functions of NF-κB and IRF via the JAK/STAT signalling cascade, attaches to the enhancer A region and the interferon-stimulated response element (ISRE) of the MHC-I promoter region, upsurges the expression of genes such as MHC-I, B2M, and TAP transporter proteins, to amplify the antigen-presenting capacity. Nevertheless, mutation in genes affiliated with the IFN-γ/JAK/STAT signaling pathway have been identified in a wide spectrum of cancer, giving rise to antigen presentation impairments and resistance to ICIs treatment (45, 46). A plausible explanation for this phenomenon is that the compromised IFN-γ/JAK/STAT signaling axis instigates the inactivation of the histone dimethyltransferase WHSC1, thereby restricting the expression of B2M/MHC-I (47). On the contrary, non-coding RNA (ncRNA) is proficient in curbing the expression of genes associated with the antigen presentation pathway. In colorectal cancer, miR-148a-3p is eminently capable of repressing the expression of calnexin (CANX) and B2M/MHC-I, thereby decidedly restricting the anti-cancer efficacy of CTLs (48). Additionally, in esophageal cancer, miR-148-3p and miR125a markedly depress the expression of MHC-I and B2M, conspicuously curtailing the overall survival of cancer patients (49).

## 4 Mechanisms of resistance to B2M-related immunotherapy

In 2020, the Society for Immunotherapy of Cancer (SITC) delineated primary and secondary resistance to ICIs therapy for advanced tumors (50). The ascertainment of the mechanism of tumor immune resistance and the sifting of the beneficiary population are among the urgent problems to be tackled in the contemporary medical era. Presently, the mechanisms of tumor immune resistance consist of: (1) intrinsic resistance mechanisms; (2) extrinsic resistance mechanisms; (3) host-related resistance mechanisms. Among them, the mechanism of immunotherapy resistance related to B2M is mainly manifested as antigen presentation impairment (51, 52). that is, B2M reduction/defect impairs the proper assembly and transport of MHC-I molecules to the cell surface. As a result, CD8^+^ T cells fail to recognize the “danger signals” presented on the tumor cell surface, thereby preventing the initiation of an effective immune response. This phenomenon effectively renders tumor cells “invisible” to the immune system, allowing them to evade immune surveillance and elimination, ultimately promoting tumor growth, metastasis and resistance. Prior investigations have revealed that B2M mutation may aid tumor cells to break away from the normal immune response and attenuate the efficacy of CTLs based immunotherapies by impeding MHC class I-mediated tumor antigen presentation (27, 30). Among melanoma patients undergoing anti-PD-1 treatment, Sade-Feldman et al. (34)ascertained that B2M LOH in non-responders was threefold that in responders. Furthermore, IFN-γ potentiates CTLs activity by inducing upregulation of MHC-I complex and concurrently elicits a robust tumor-killing response via pro-apoptotic and anti-proliferative effects. However, the existence of genetic mutation and defect affiliated with the IFN-γ/IFNGR/JAK/STAT pathway can also give rise to resistance against ICIs. It is worth mentioning that recent research has shown that inhibiting autophagy can restore B2M/MHC-I expression and improve antigen presentation, thereby enhancing anti-tumor T cell responses (53), indicating that autophagy pathway can also promote immune escape by degrading B2M/MHC-I, indirectly contributing to immune resistance.

## 5 Correlation between B2M and immunotherapy resistance based on scRNA-seq and IMC

scRNA-seq represents an advanced technology that facilitates high-throughput transcriptomic analysis at the single-cell level. This approach not only identifies novel cell types and rare cell populations but also provides precise insights into tumor heterogeneity, the intricate interactions between tumor cells and their microenvironment, and the detailed evolutionary trajectories of individual tumors, so as to further explore the mechanism of tumor immunotherapy resistance (54, 55). IMC compensates for the lack of tissue spatial information in scRNA-seq and effectively resolves the serious cross-color issue among fluorescent groups. Building upon *in situ* immunohistochemistry (IHC), this technology integrates cytometry by time of flight (CyTOF) and laser ablation techniques, allowing for the simultaneous detection of abundance variations and spatial distribution of up to 50 targets within tissue samples at subcellular resolution, thereby generating single-cell proteome map across temporal and spatial dimensions (56, 57). Owing to its distinctive technical merits, IMC has emerged as a powerful tool in the realm of tumor research. Utilizing scRNA-seq and IMC technologies to unravel the correlation between B2M and immunotherapy resistance offers valuable insights for overcoming resistance and devising innovative therapeutic strategies (**Table 1**).

**Table 1:** Summary of publications on B2M and immunotherapy resistance based on scRNA-seq and IMC.

Ref No.	Research technique	Cancer type	Target	Cells (n)	Patients (n)	Main finding
Kim et al. (58)	scRNA-seq CyTOF WES IHC FC	LUAD	tS2 B2M	208,506 cells	44	The study identified a cancer cell subtype tS2 that deviated from the normal differentiation trajectory and dominated metastasis, revealing that remodeling of tumor-derived vascular endothelial cell subsets reduced antigen presentation (including B2M) and immune cell homing activity, providing new insights into understanding cellular behavior and tumor immune escape mechanisms in lung cancer TME.
Liu et al. (59)	scRNA-seq Bulk RNA-seq FISH FACS/IHC FC	OS	HLA-A HLA-B HLA-E B2M CD24	–	GSE152048 GSE162454 85	The downregulation of MHC-I and B2M diminished immunogenicity in high-grade OS, which may be a potential mechanism for cancer immunotherapy resistance. In addition, CD24, a novel “don’t eat me” signal, also facilitated immune evasion of osteosarcoma cells.
Michele et al. (60)	IMC mIF WES RNA-seq TCR-seq IHC	CRC	B2M MHC-I Wnt IFN-γ TMB CD74 CD3 CD8	20,890 T cells 16,748 macrophages	29	In CRCs, low TMB served as a marker of resistance, which was considered to be associated with higher activation of the Wnt pathway leading to immune microenvironment of “cold tumors”. Additionally, the correlation of B2M deficiency with immune resistance and overall low PD-1 and PD-L1 expression, were specific characteristics of CRCs.
Sandra et al. (61)	IMC QIF DSP	Melanoma	B2M MHC-I LAG3 CD8 CD4 CD3 CSF1R PD-1	–	312	This research confirmed the reliability and effectiveness of the AQUA platform in IMC analysis, and found that high B2M expression was associated with better immunotherapy response and longer PFS and OS based on IMC, QIF and DSP technologies, revealing that B2M has significant potential as a biomarker for evaluating the efficacy of immune therapy.
Natasja et al. (64)	scRNA-seq Bulk RNA-seq WGS IMC IHC FC CRISPR/Cas9	MMR-d cancers (CRC, STAD, UCEC, COAD)	B2M CD103 CD39 GZMB Ki-67 CD4 KIR	4,442 γδ T cells	TCGA: 239 DRUP: 71 Hartwig: 2,256 NICHE:10 IMC:17 PDTOs:2	γδT cells can function as critical effectors in ICIs therapy against HLA-I/B2M-deficient cancers, representing a novel mechanism and therapeutic strategy for monitoring tumor immune escape.
Gurjao et al. (80)	scRNA-seq Bulk RNA-seq WES IHC MIF	dMMR /MSI-H CRC	B2M	595 cells	2	The biallelic loss of B2M (frameshift deletion/loss of heterozygosity) leads to impaired antigen presentation, which may contribute to intrinsic resistance to immune checkpoint blockade therapy in dMMR/MSI-H colorectal cancer. Furthermore, NK cell-based immunotherapies, particularly those involving the adoptive transfer of “educated” NK cells, represent a promising alternative therapeutic strategy.
Song et al. (44)	scRNA-seq FC WB qRT-PCR	MCC	B2M TAP1 LMP2 HLA-A Caspase-3/7	10,816 cells	–	The HDAC inhibitor Domatinostat promotes cell cycle arrest, induces apoptosis and upregulates APM genes and MHC-I/B2M expression to reverse immune resistance.
Kypraios et al. (62)	scRNA-seq hdWGCNA	T-ALL	B2M HLA-A HLA-B	–	3	This study identified multiple genes associated with recurrence, such as HLA-A/B and B2M, through scRNA-seq and gene co-expression networks analysis and on paired diagnosis-relapse samples, indicating a potential role in immune interference and contributing to resistance.
Zhang et al. (63)	scRNA-seq WES IHC	SCLC	MHC-I B2M FZD8	24,081 cells	7	In SCLC, antigen processing and presentation of peptide antigens via MHC-I related genes (B2M) were less activated in malignant cells of the patients with lower immune infiltration than those with higher immune infiltration, which is of great significance for elucidating the mechanisms of tumor resistance.

scRNA-seq, Single cell RNA sequencing; CyTOF, Cytometry by time-of-flight; WES, Whole-exome sequencing; IHC, Immunohistochemistry; FC, Flow cytometry; LUAD, Lung adenocarcinoma; TME, Tumor microenvironment; FISH, Fluorescence *in situ* hybridization; FACS, Fluorescence-activated cell sorting; OS, Osteosarcoma; IMC, Imaging mass cytometry; mIF, Multiplex immunofluorescence; TCR-seq, T-cell receptor sequencing; CRC, Colorectal cancer; TMB, Tumor mutational burden; QIF, Quantitative immunofluorescence; DSP, Digital spatial profiling; WGS, Whole genome sequencing; STAD, Stomach adenocarcinoma; UCEC, Uterus corpus endometrium carcinoma; COAD, Colorectal adenocarcinoma; PDTOs, Patient-derived tumor organoids; dMMR/MSI-H, Deficient mismatch repair/microsatellite instability-high; NK, Natural killer; WB, Western blot; qRT-PCR, Quantitative real-time polymerase chain reaction; MCC, Merkel cell carcinoma; HDAC, Histone deacetylase; APM, Antigen processing and presentation machinery; hdWGCNA, High-dimensional weighted gene co-expression network analysis; T-ALL, T cell acute lymphoblastic leukemia; SCLC, Small cell lung cancer.

In May 2020, the Samsung Medical Center (SMC) team conducted scRNA-seq on tumor samples from 44 patients with lung adenocarcinoma (58). This study not only identified a cancer cell subtype tS2 closely associated with lung adenocarcinoma metastasis but also uncovered that the reprogramming of tumor-derived vascular endothelial cell subsets in lung adenocarcinoma patients impaired antigen presentation (including B2M) and the homing activity of immune cells (58). These findings provide new insights into understanding cell behavior and tumor immune evasion mechanisms within the tumor microenvironment (TME) of lung adenocarcinoma. The team led by Weijian Liu in China utilized scRNA-seq technology to characterize the immunosuppressive tumor microenvironment profile of osteosarcoma (OS) and discovered that MHC-I (HLA-A, HLA-B, and HLA-E)/B2M genes were downregulated, indicating diminished tumor immunogenicity in OS (59). To further investigate whether the downregulation of MHC-I/B2M is prevalent in OS, they assessed the expression of MHC-I/B2M in OS patient samples by IHC. The results confirmed that high-grade OS indeed exhibited downregulation of MHC-I/B2M. Based on these findings, we propose that the downregulation of MHC-I/B2M in high-grade OS may contribute to resistance to immune therapy. What’s more, several scholars conducted a comprehensive analysis of 738 regions with varying degrees of T-cell infiltration from 29 colorectal cancer patients using multiple detection techniques (WES, TCR-seq, RNA-seq, IMC/mIF). They discovered that B2M expression in the tumor parenchyma was lower in non-responsive groups compared to responsive groups, while no significant difference was observed in the tumor stroma (60). Sandra et al. (61) analyzed formalin-fixed, paraffin-embedded (FFPE) tissue microarray (TMA) samples from patients with metastatic melanoma who received PD-1 blockade therapy using IMC technology. Their findings indicated that higher B2M expression correlated with better immune therapy response and longer progression-free survival (PFS) and overall survival (OS). Collectively, these studies implied that silent mutation and loss of B2M are linked to immunotherapy resistance (62, 63), while upregulating B2M may offer a new therapeutic strategy for overcoming resistance. However, Natasja and his colleagues isolated γδT cells from 5 mismatch repair-deficient (MMR-d) colon cancer tissue for scRNA-seq, revealing that PD-1, activation, proliferation and killer genes expressed by γδ1 and γδ3 T cells were significantly upregulated in B2M-deficient tumors (64). Additionally, they performed IMC analysis on 17 MMR-d colon cancer patients who had not undergone ICB treatment. The results showed a notable increase in the infiltration of γδT cells in B2M-mutant tumors, with these cells exhibiting high intraepithelial localization characterized by significant expression of CD103, CD39, GZMB, Ki-67, and PD-1. Subsequently, they established two patient-derived tumor organoid cell lines and generated B2M knockout (B2M KO) cell lines using CRISPR technology. The authors exposed these B2M KO and parental B2M wild-type (B2M WT) counterparts to expanded γδ T cell subsets and quantified γδ T cell activation by measuring IFNγ expression, finding that γδT cells exhibited greater reactivity against the B2M KO cell lines compared to B2M WT cell lines. These findings unveil that γδT cells can serve as crucial effectors in ICIs therapy for combating HLA-I/B2M-deficient cancers, which represent a novel mechanism and therapeutic approach for monitoring tumor immune resistance.

## 6 Correlation between B2M and immunotherapy resistance based on CRISPR/Cas9

The CRISPR/Cas9 system, hailed as a revolutionary “gene scissors” technology, is renowned for its flexibility, convenience, high throughput, efficiency, and precision. It has found extensive application in various fields, including tumor functional gene screening, signal pathway analysis, and resistance target discovery. The CRISPR-Cas9 system comprises two components: Cas9 nuclease and single guide RNA (sgRNA). The sgRNA plays a critical role in recognizing specific genomic sequences. Upon complementary base pairing with the target DNA sequence, the Cas9 protein, which possesses endonuclease activity, cleaves the DNA at the specified site. Following cleavage, the broken DNA undergoes repair via either non-homologous end joining (NHEJ) or homology-directed repair (HDR), enabling genome editing, insertion, or defect (65). Utilizing CRISPR/Cas9 high-throughput gene screening technology, it is possible to establish stable cell lines with targeted gene knockouts (**Table 2**), thereby providing an excellent platform for screening resistance targets (66).

**Table 2:** Summary of studies on B2M and immunotherapy resistance using CRISPR/Cas9.

Ref No.	Location	Target	Genome-editing technology	Effects after CRISPR-engineering
Chariou et al. (67)	Cancer cell Colon cancer (MC38) Breast cancer (EMT6)	B2M LMP2 JAK1	CRISPR-KO	B2M KO impairs antigen presentation; JAK1 KO decreases PD-L1 expression; B2M/LMP2/JAK1 KO confer resistance to αPD-1 and αPD-L1 therapies.
Gettinger et al. (68)	Cancer cell lung cancer (UN-SCC680AJ)	B2M	CRISPR-KO	B2M KO induces resistance to PD-1 inhibitor therapy.
Mark et al. (38)	Cancer cell Leukemia (K-562) SCLC (NCI-H82, NCI-H146, NCI-H69) Neuroblastoma (Kelly, IMR-32) MCC (MCC-002)	EED EZH2 SUZ12 MTF2 NLRC5 MHC-1 B2M	CRISPR-KO	Core PRC2 members (EED, SUZ12, EZH2) KO significantly upregulate the expression of critical genes (B2M, MHC-I) in the MHC-I antigen processing pathway, thereby reversing T cell-mediated immune escape.
Kearney et al. (69)	Cancer cell Colon cancer (MC38)	B2M TAP1 JAK1 STAT1 Tnfrsf1a Caspase-8 ADO	Genome-wide immune evasion screening	Knockout of crucial genes (Caspase-8, Tnfrsf1a, ADO, JAK1, STAT1, B2M, TAP1) in the TNF signaling, IFN-γ signaling, and antigen presentation pathways serve as a key determinant of resistance to CD8^+^ T cell and NK cell-mediated cytotoxicity.
Wang et al. (66)	Cancer cell HCC (MAL1) Bladder cancer (MB49) Melanoma (B16-F10) TNBC (E0771)	KMT2D TP53 B2M Grif1 BCOR	CRISPR-KO	KMT2D KO potentiates the anti-tumor efficacy of ICIs by inducing DNA damage, augmenting mutational burden, enhancing IFN-γ-stimulated antigen presentation, and promoting infiltration of PD-1^+^ T cells and macrophages. Additionally, comparing anti-PD-1 treated mice with PBS treated mice, B2M KO was correlated with anti-PD-1 resistance.
Dufva et al. (72)	Cancer cell BCL (SUDHL4) B-ALL (NALM6) MM (MM1.S, LP1, KMS11) CML (K562) AML (MOLM14)	B2M TAP1 TAP2 MHC-I	CRISPR-KO	Knockout of antigen-presenting genes, including MHC-I and B2M, enhances the sensitivity of across hematologic cancer cell lines to NK cells, supporting the “missing-self” mechanism of NK cell activation.
Torrejon et al. (73)	Cancer cell Colon cancer (MC38) Melanoma (B16-F10, YUMMER2.1)	B2M	CRISPR-KO	In the context of B2M KO, the activation of CD4^+^ T cells and NK cells can overcome resistance to PD-1 blockade therapy to enhance anti-tumor efficacy.
Germano et al. (74)	Cancer cell Colon cancer (MC38, CT26)	B2M	CRISPR-KO	In the context of B2M KO, the activation of CD4^+^ T cells can overcome resistance to ICIs treatment.
Freeman et al. (71)	Cancer cell Melanoma (B16-F10)	B2M TAP1 TAP2 JAK1 JAK2 Ifngr2	CRISPR-KO	The immune escape from T cells caused by the knockout of genes IFN-γ signaling pathway (JAK1, JAK2, Ifngr2) and antigen presentation pathway (TAP1, TAP2, B2M) promotes tumor vulnerability to NK cells.
Dubrot et al. (70 *)*	Cancer cell Renca Renal Cell Line	Atg5 B2M H2-T23 TAP1 TAP2	CRISPR-KO	Knockout of antigen presentation genes (H2-T23, TAP1, TAP2, B2M) and the autophagy gene Atg5 can significantly enhance NK cell-mediated immune responses, overcome immune resistance, and sensitize tumors to immunotherapy.
Kobayashi et al. (37)	Cancer cell Melanoma (B16-F10) Breast cancer (MCF7)	NLRC5 MHC-I B2M TAP1	Modified CRISPR/Cas9 system (TRED-1)	Targeting NLRC5 promoter demethylation through TRED-I system can upregulate MHC-I, B2M, TAP1 and genes encoding immune proteasome components (LMP2/PSMB9/β1i, LMP7/PSMB8/β5i), thereby enhancing CD8^+^T cell-mediated anti-cancer immunity.
Eyquem et al. (96)	CAR-T cell	TRAC B2M	CRISPR-KO	TRAC/B2M KO reduces the probability of initiating GvHD and eliciting donor T cell rejection, thereby enhancing the effectiveness of CAR-T therapy.
Choi et al. (97)	CAR-T cell	TARC B2M PD-1	CRISPR-KO	TRAC/B2M/PD-1 KO significantly enhances the anti-tumor efficacy of EGFRvIII CAR T cells.
Ren et al. (94)	CAR-T cell	TCR B2M PD-1	CRISPR-KO	B2M/TCR/PD-1 KO can reduce immunogenicity, prevent GvHD, and significantly enhance the anti-tumor activity of CAR-T cells.
Chen et al. (89)	CAR-T cell	TCR B2M	CRISPR-KO	TCR/B2M KO eliminates GvHD while enhancing the anti-tumor efficacy of CAR-T cells.
Pavlovic et al. (93)	CAR-T cell	TRAC B2M	CRISPR-KO	TRAC/B2M KO reduces GvHD and concurrently enhances the efficacy and safety of allogeneic CAR-T cells.
Liu et al. (92)	CAR-T cell	TRAC B2M PD-1	CRISPR-KO	TRAC/B2M/PD-1 KO eliminates GvHD and significantly enhances the anti-tumor efficacy of CAR-T cells.
Guo et al. (90)	CAR-T cell	TCR B2M	CRISPR-KO	TCR/B2M KO reduces GvHD and enhances the efficacy of universal CAR-T cells in patients with relapsed/refractory lymphoma.
Kagoya et al. (91)	CAR-T cell	B2M CIITA TRAC	CRISPR-KO	B2M/CIITA/TRAC KO avoids inducing GvHD and enhances the anti-tumor effect of CD19 tKO CAR-T cells

KO, Knockout; SCLC, Small cell lung cancer; MCC, Merkel cell carcinoma; PRC2, Polycomb repressive complex 2; NK, Natural killer; HCC, Hepatocellular carcinoma; TNBC, Triple negative breast cancer; ICIs, Immune checkpoint inhibitors; PBS, Phosphate buffered saline; BCL, B-cell lymphoma; B-ALL, B cell acute lymphoblastic leukemia; MM, Multiple myeloma; CML, Chronic myeloid leukemia; AML, Acute myeloid leukemia; CAR, Chimeric antigen receptor; GvHD, Graft-versus-host disease; EGFR, Epidermal growth factor receptor.

Previous studies have successfully utilized CRISPR/Cas9 technology to establish knockout models of B2M, JAK1, and LMP2 in the mouse EMT6 breast cancer cell line, as well as B2M knockout clone model in the mouse MC38 colon cancer cell line. *In vivo* experiments demonstrated that mice bearing tumors derived from B2M (EMT6 and MC38) or Jak1/LMP2 (EMT6) knockout single-cell clones exhibited no response to αPD-1 or αPD-L1 treatment (67). Similarly, Gettinger et al. (68) employed CRISPR technology to knock out B2M in mouse lung cancer cells. Wild-type and B2M-knockout cells were injected into the right legs of immunocompetent A/J mice. When tumor volumes reached approximately 30 mm³, the mice were randomly assigned to receive anti-PD-1 or isotype control antibodies. The results indicated that mice harboring intact B2M responded to PD-1 antibody treatment, whereas those with B2M KO tumors showed no therapeutic benefit from PD-1 blockade. Furthermore, Kearney et al. (69) performed a series of genome-wide screens using a CRISPR/Cas9-based custom immune escape library in colon cancer mouse models. They identified that the absence of crucial genes (caspase-8, JAK1, STAT1, B2M, TAP1) in TNF signaling, IFN-γ signaling, and antigen presentation pathways conferred resistance to CD8^+^ T cell and NK cell-mediated cytotoxicity. In conclusion, these findings underscore the significant part of B2M deficiency in mediating immunotherapy resistance. However, several studies have reported that high immunogenic cancer patients with B2M defect can still exhibit durable responses to anti-PD-1 therapy, suggesting the involvement of immune cell subsets beyond CD8^+^ T cells in these responses (70, 71). The collaborative work of Satu Mustjoki’s team from the University of Helsinki and Constantine S. Mitsiades’ team from Harvard University utilized genome-scale CRISPR knockout (LOF) screens to co-culture transfected tumor cells with expanded NK cells for periods ranging from 24 hours to 2 weeks. They observed that knocking out antigen presentation genes, including MHC-I and B2M, enhanced the sensitivity of all blood cancer cell lines to NK cells, supporting the “missing self” activation mechanism of NK cells (72). Additionally, Torrejon et al. (73) knocked out B2M gene in three mouse tumor cell lines (MC38, YUMM2.1 UV, and B16) with varying baseline MHC-I expression and sensitivities to anti-PD-1 therapy by CRISPR/Cas9 technology. Using the OMIQ platform and CyTOF, they characterized tumor immune infiltration and found that MC38 and YUMMER2.1 cells with B2M KO responded to anti-PD-1 treatment alone or in combination with IL-2 agonists, mediated by CD4^+^ T cells and NK cells. In contrast, more aggressive B16 cells lacking B2M showed only a partial response to IL-2 agonists, dependent on NK cells. Equally, Germano and his team also used CRISPR-Cas9 to construct B2M KO MMRd colorectal cancer cell lines (MC38-MMRd-B2M-/-and CT26-MMRd-B2M-/-) (74), subcutaneously inoculated the cells into C57BL/6 mice, and administered anti-PD-1 and anti CTLA-4 antibodies and found that B2M-deficient loaded mice treated with ICIs showed significant tumor regression and that in CD8^+^ T-cell depleted mice, ICIs remained efficacious against MMRd B2M-deficient tumors. Conversely, ICIs failed to induce tumor regression in CD4^+^ T cell-depleted mice. Additionally, they discovered a correlation between low B2M expression and increased CD4^+^T cell infiltration in tumors, strongly indicating that CD4^+^T cells mediate the response to B2M-deficient tumors during ICIs treatment. Evidently, it can be seen that in the context of B2M deficiency, the activation of CD4^+^ T cells and NK cells can also overcome resistance to exert anti-tumor functions.

## 7 Association of B2M with different immunotherapy resistance and reversal strategies

**Figure 2:**
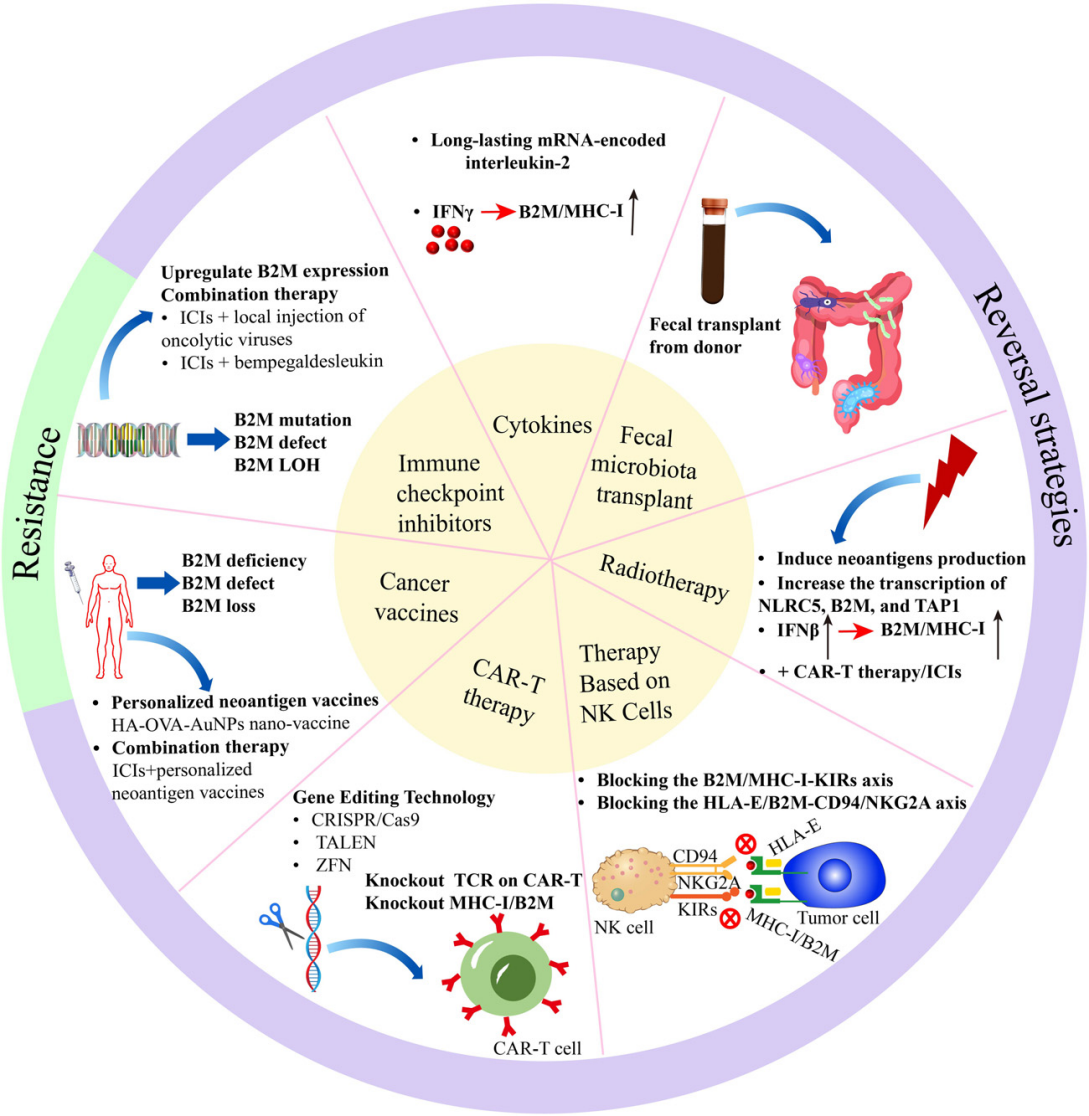
The association of B2M with immunotherapy resistance and reversal strategies.

Immunotherapy brings new hope to cancer patients, yet it encounters the challenge of resistance. B2M, a pivotal factor in the immune regulatory network, plays a crucial role in immunotherapy resistance. A thorough investigation into the association between B2M and different immunotherapy resistance, along with identifying effective strategies to reverse resistance caused by B2M defect (**Figure 2**), can not only significantly improve the clinical efficacy of immunotherapy but also potentially pave new avenues for overcoming resistance in cancer immunotherapy.

### 7.1 ICIs

B2M is a crucial molecule indispensable for the assembly of MHC-I complexes and tumor antigen presentation, and its defect might be a prevailing cause of resistance to ICIs therapy. Relevant research demonstrates that B2M LOH is prevalently observed among cancer patients who exhibit poor or no response to ICIs (75). B2M defect induces resistance in ICIs-treated lung cancer patients, while upregulation of B2M enhances the efficacy of immunotherapy (76). An association could be found in a cohort study between elevated B2M expression and superior immune responses and significantly prolonged survival in metastatic melanoma patients undergoing ICIs treatment (61). Recently, acquired resistance to pembrolizumab was reported in a patient with advanced melanoma, which might be attributed to B2M defect (28). Results from the study showed that B2M truncating mutation occurred in this patient, resulting in antigen presentation defect to induce immune resistance. Similarly, Wei et al. (77) reported a 27-year-old female patient with stage IIIB (pT3N2aM0) colorectal cancer characterized as MSI-H/dMMR who developed resistance after two months of anti-PD-1 therapy. Next-generation sequencing (NGS) analysis revealed an increase in TMB to 60 muts/MB and an MSH2 mutation (p.Q337*) detected in the tumor tissue DNA of the patient. Additionally, INDEL mutation (c.43_44del) was found at the microsatellite site of the B2M gene exon, resulting in the expression of a B2M chimeric protein p.L15Ffs*41. These results suggest that B2M mutation may contribute to ICIs resistance. It is worthy of note that the intensive and persistent T cell selection pressure generated by ICIs can conversely promote B2M mutation. It was observed in lung cancer patients undergoing chemotherapy in combination with ICIs treatment that B2M experienced heterozygous loss during chemotherapy and complete loss subsequent to ICIs (68). Nevertheless, some studies have reported that 85% of B2M mutant colorectal cancer patients can benefit from ICIs, manifested as disease stability or partial remission (78). In a study conducted by Professor Peirong Ding’s team, involving 35 MSI-H CRC patients receiving PD-1 therapy, no statistically significant difference was observed in the efficacy between patients with B2M mutation and those with wild-type B2M (*p*=0.53). This suggests that MSI-H CRC patients harboring B2M mutation are also potential beneficiaries of PD-1 antibody therapy (79). Considering these findings, we speculate that CTLs antigen recognition becomes compromised due to B2M mutation/deletion, while other immune cells such as CD4^+^ T lymphocytes, NK cells, and γδ T cells may continue to exert cytotoxic effects on tumor cells (64, 70–74, 80). Unfortunately, there are currently limited studies on B2M, and the precise mechanisms by which these alternative immune cells are activated and mediate immune responses in the setting of B2M deficiency warrant further investigation.

The combination therapeutic strategies have been accorded precedence and undergone extensive exploration aimed at reversing the resistance to ICIs elicited by B2M mutation/defect. Local injection of oncolytic viruses holds the potential to overcome ICIs resistance attributed to B2M mutation and enhances anti-tumor responses through activating the IFN-γ/JAK/STAT signaling axis. B2M frameshift mutation was discerned in a patient with intractable stage IV metastatic melanoma after receiving the combination therapy of ipilimumab and nivolumab. Subsequently, the patient underwent sequential treatment with transgenic oncolytic virus TVCE in conjunction with pembrolizumab and temozolomide, interestingly, the metastatic lesions achieved a protracted (19 months) complete response (81). Furthermore, bempegaldesleukin (NKTR-214, an immunostimulatory IL-2 prodrug), which is competent in activating and continuously expanding CD4^+^ T and CD8^+^ T cells, manifests a synergistic anti-tumor effect when combined with ICIs. Systemic administration of bempegaldesleukin can overcome resistance incurred by anti-PD-1 treatment and achieve better survival in knockout B2M melanoma mice (82).

### 7.2 Cancer vaccines

Cancer vaccines are biological agents used for the prophylaxis or therapeutics of tumors, in which tumor antigens are introduced into the patient’s body in various forms (such as nucleic acids, protein polypeptides, bacterial and viral vectors, DC cells) to activate specific immune responses and establish long-term immune memory. They possess crucial clinical significance on account of the merits of high efficacy, strong specificity, favorable safety, ae well as long-term immune memory. Nevertheless, cancer vaccines based on T cells may develop resistance because of B2M defect. Benitez’s study discovered that two melanoma patients who were refractory to MAGE-peptide tumor vaccine treatment with loss of B2M expression (83). The poor efficacy of cancer vaccination administration with ODN1826 adjuvant in MHC-I negative models may be affiliated with B2M deficiency in TC-1 cell lines (84).

Presently, personalized neoantigen vaccines have inaugurated a novel epoch for cancer vaccines. Neoantigens are exclusively expressed by tumor cells and can evoke a bona fide tumor-specific T-cell response, thereby evading “off-target” damage to non-tumor tissues. Moreover, neoantigens are nascent epitopes originated from somatic mutations and can avoid central immune tolerance, thus possessing a high immunogenicity. Tumor neoantigen vaccines can potentiate immune response elicited by neoantigen-specific T cells, surmount immune escape, and forge new avenues for achieving precise immunotherapy. Cao et al. (85)elaborated the HA-OVA-AuNPs nano-vaccine, which enlisted near-infrared (NIR) irradiation for photothermal regulation of cytoplasmic antigen delivery to potentiate downstream MHC-I antigen presentation. Campo et al. (86) transfected the adenovirus vector harboring B2M gene (AdCMV*B2M*) into MHC-I negative/B2M-deficient malignant tumor (melanoma, colorectal cancer, prostate cancer) cell lines. It was ascertained that the expression of HLA-I/B2M was positive after 72 hours, escalating the immunogenicity of tumor cells and the capability of T cell immune recognition. Additionally, enhanced clinical responses have been procured in the combined treatment of cancer vaccines and ICIs, which has been validated in clinical studies. In 2020, a phase IIb clinical trial, NCT02897765, was the first to incorporate the personalized neoantigen vaccine NEO-PV-01 with PD-1 inhibitors for NSCLC, advanced melanoma or bladder cancer, which demonstrated that all subjects exhibited neoantigen-specific CD4^+^ and CD8^+^ T cell responses with conspicuously higher objective response rate (ORR) than that of monotherapy (87).

### 7.3 CAR-T therapy

CAR-T therapy, a novel adoptive cell immunotherapy that has garnered considerable attention in recent years, utilizes genetic engineering techniques to activate T cells and empower them to express chimeric antigen receptors (CAR). Through amplifying CAR-T cells ex vivo, it overcomes the local immunosuppressive microenvironment and disrupts the host immune tolerance, targeting and eliminating tumor cells in a non-MHC-restricted manner. Currently, autologous CAR-T therapy has attained remarkable breakthroughs in the realm of immunotherapy for B-cell malignant lymphoma (88). Nevertheless, against the backdrop of autologous T cell therapy that necessitates customization, prolonged preparation cycle (approximately 2-3 weeks), limited quantity, and suboptimal quality, allogeneic CAR-T cell therapy has emerged. The latter, also known as universal CAR-T and off-the-shelf CAR-T, pertains to the procedure where T cells are extracted from healthy donors or obtained through means such as peripheral blood, umbilical cord blood, or pluripotent stem cells, subjected to genetic engineering modification and amplification, and ultimately transferred *in vivo* to exert the predefined anti-tumor effect. However, graft-versus-host disease (GvHD) and host-versus-graft reaction (HvGA) remain two major obstacles for allogeneic CAR-T therapy.

In recent years, gene editing technologies (CRISPR/Cas9, TALEN, ZFN) have contributed to addressing GvHD and HvGA by knocking out TCR on allogeneic CAR-T along with MHC-I/B2M, thus improving the anti-tumor effectiveness of CAR-T cells (89–94) (**Table 2**). A study (95) utilized the zinc finger nuclease (ZFN) system, in combination with the Sleeping Beauty transposase/transposition system, to engineer CAR-T cells that are CD19-specific and lack endogenous TCR expression. Eyquem et al. (96) utilized CRISPR/Cas9 technology to target the disruption of endogenous T cell receptor (TRAC) and B2M, thereby minimizing the probability of initiating GvHD and eliciting donor T cell rejection, and subsequently enhancing the efficacy of CAR-T therapy. In 2019, Choi et al. (97) exploited the CRIPSR/Cas9 system to perform multiple gene disruptions of TARC, B2M, and PD-1 (PDCD1), creating allogeneic epidermal growth factor (EGFRvIII) CAR-T cells that can resist PD-1 inhibition. The results demonstrated that the EGFRvIII CAR-T therapy conspicuously augmented the anti-tumor activity in the preclinical model of glioblastoma (GBM) and significantly ameliorated the survival of the mice model. Nevertheless, it is worth noting that B2M/MHC-I deficiency might trigger the activation of natural killer (NK) cells, which would recognize the allogeneic CAR-T cells as “missing self” and exert immune rejection (98). Hence, additional research is requisite restrain or eradicate the reactive NK cells within the host, thereby offering protection to universal CAR-T cells against being killed by NK cells.

### 7.4 Therapies based on natural killer cells

NK cells, otherwise designated as natural killer cells, can nonspecifically eliminate tumor cells without prerequisite stimulation of tumor antigens. Their killing activity is not circumscribed by MHC and is independent of antibodies, predominantly hinging upon the intricate interaction of activating and inhibitory receptors on their surface. The inhibitory receptors existing on the surface of NK cells, such as killer immunoglobulin-like receptors (KIRs) and Natural Killer Group 2 Member A (NKG2A), are capable of recognizing MHC-I molecule, thereby preventing NK cell activation, polarization, and degranulation. Nevertheless, tumor cells typically exhibit low or no expression of B2M/MHC-I, engendering the deprivation of the B2M/MHC-I/KIRs interaction and subsequently inducing NK cell activation, a process denominated as “missing self-recognition.” A study revealed that, the downregulation of B2M expression (approximately 9.8%) among NSCLC patients was correlated with an augmented infiltration of NK cells (99). Abnormal overexpression of HLA-E/B2M was found to be associated with NKG2A-expressing CD94^+^ T cells and NK cells in MSI colorectal cancer, and an increased number of NKG2A^+^CD94^+^ T cells was interrelated with an unfavorable prognosis (100). These findings imply that the HLA-E/B2M-CD94/NKG2A axis may partake in tumor immune escape, and corresponding strategies to block B2M/NKG2A may usher in novel prospects for NK cell-based immunotherapy for cancer patients.

### 7.5 Radiotherapy

Radiotherapy can induce immune activation and elicit anti-tumor responses through mediating DNA damage in tumor cells, reshaping tumor immune microenvironment like an *in-situ* vaccine, and initiating the release of inflammatory factors, which may potentially confer benefits upon cancer patients who are resistant to ICIs again. It has been found that radiotherapy can facilitate the release of interferon-beta (IFNβ) to upregulate B2M/MHC-I expression on resistant tumor cells, enhance antigen presentation, and restore responsiveness to PD-1 therapy (101). It can also significantly increase the transcription of NLRC5, B2M, and TAP1 in a dose-dependent manner to overcome resistance to ICIs (102). Moreover, A synergy is attained when radiotherapy is combined with CAR-T therapy in a mouse glioblastoma model, and the underlying mechanism could be attributed to the circumstance that radiotherapy potentiates recognition and cytotoxicity of CD8^+^ T cells through upregulating B2M/MHC-I (103). The combinations of Stereotactic radiotherapy (SBRT) with camrelizumab brought pronounced survival benefit in overall survival (OS) and progression-free survival (PFS) for unresectable hepatocellular carcinoma patients, attributed to the “abscopal effect” of radiotherapy, as demonstrated by one prospective single-arm clinical trial (104). However, the optimal radiotherapy dose and fractionation are still under vigorous exploration to evoke systemic anti-tumor immune responses. On the other hand, radiotherapy can also induce the production of neoantigens to circumvent inadequate antigen presentation and promote CTLs infiltration to boost immunogenicity. Collectively, radiotherapy holds the potential to serve as a promising alternative for reversing immune resistance in B2M/MHC-I-deficient cancer patients.

### 7.6 Other therapies

Mounting evidence suggests that altering the gut microbiota has turned to be a promising approach to reverse resistance across a variety of cancer patients. NCT03772899, a Phase 1 clinical trial, demonstrated that fecal microbiota transplant (FMT) as a latent strategy for overcoming ICIs resistance in patients with advanced melanoma (105). V. Gopalakrishnan’s team discovered that the diversity of gut microbiota was higher in the anti-PD-1 treatment responsive group (R) than in the non-responsive group (NR) (106). It was found that the abundance of fecal bacteria for R group was conspicuously elevated in the FMT experiment of sterile mice, particularly with a marked enrichment of Bacteroides. Moreover, the density of CD8^+^ T cells in the tumor tissues of mice that received FMT from the R group was higher than that of mice receiving NR transplant. Notably, the role of cytokines can’t be overlooked as immune modulators to markedly ameliorate ICIs efficacy for cancer patients. IFNγ can facilitate CD8^+^ T cell priming and infiltration by upregulating the expression of B2M/MHC-I on the surface of tumor cells. The Beck research team, by establishing a B2M-deficient mouse tumor model, revealed that MHC-I deficiency would result in severe immune desertification in TME and widespread resistance to ICIs. Long-lasting mRNA-encoded interleukin-2 (IL-2) can boost potent and durable antitumoral immune responses against B2M/MHC-I-deficient tumors, restore immune cell infiltration, and exhibit highly pro-inflammatory TME, thereby overcoming resistance incurred by ICIs treatment (107).

## 8 Conclusion and prospects

Up to now, immunotherapy has assumed a pivotal role and emerged as a milestone breakthrough in the history of tumor treatment. Nevertheless, immune resistance, to a certain extent, restricts its clinical application and constitutes a “stumbling block” for the long-term benefit for each patient with cancer. It is an urgently pressing and hot issue to elucidate the mechanism of immunotherapy resistance and explore novel therapeutic targets. Among these, B2M mutation/defect, which leads to antigen presentation dysfunction of MHC class I molecule, constitutes an important cause of immunotherapy resistance. Therefore, in-depth investigation into the role of B2M in tumor immunotherapy resistance and the development of corresponding strategies are of paramount importance.

This review systematically elaborates on the role and reversal strategies of B2M in tumor immunotherapy resistance from multiple perspectives. Notably, we have leveraged emerging technologies such as scRNA-seq, IMC, and CRISPR/Cas9 to deeply analyze the association between B2M and tumor immune therapy resistance. As the light chain of MHC-I molecule, B2M plays a pivotal role in tumor antigen presentation, with its expression modulated by diverse mechanisms such as genetics, epigenetics, cytokines, and non-coding RNAs. B2M mutation/defect can impair antigen presentation, which constitutes a significant factor of tumor immunotherapy resistance. Interestingly, during ICIs treatment, immune cells such as CD4^+^ T cells, NK cells, and γδ T cells mediate responses to B2M-deficient tumors, offering new avenues to overcome the resistance stemming from B2M deficiency. Additionally, combination therapies (such as oncolytic viruses/bempegaldesleukin combined with ICIs), personalized neoantigen tumor vaccines, optimized CAR-T therapy (using gene editing technology to knock out B2M), NK cell therapy, radiotherapy, fecal microbiota transplantation, and cytokine therapy also provide promising approaches to reverse immune resistance induced by B2M mutation.

Looking ahead, several critical fields in B2M-related tumor immunotherapy research warrant further exploration. Firstly, with the rapid development of high-throughput sequencing, single-cell multi-omics technology (including proteomics, transcriptomics, epigenomics, and spatial omics) can be used to comprehensively analyze the expression profiles and functional variations of B2M across diverse cell subpopulations within the tumor microenvironment. It also facilitates the precise mapping of B2M interaction profiles between tumor cells and immune cells, elucidating potential synergistic or antagonistic mechanisms, and providing a more detailed theoretical basis for precision therapy. Secondly, while CD4^+^ T cells, NK cells, and γδ T cells have been identified as key effector cells for ICB treatment in B2M-deficient cancers, the underlying mechanisms by which these cells mediate their cytotoxic effects remain to be fully elucidated. Lastly, there is a need to develop more targeted and efficacious therapies for B2M mutation/defect, such as small molecule inhibitors or biologics designed to specifically address antigen presentation defects and reverse immune resistance. In conclusion, it is imperative that we make dedicated efforts to translate these issues into practical clinical applications, thereby actualizing the concept of “from bench to bedside” possible.
